# High prevalence of Mucosa-Associated extended-spectrum β-Lactamase-producing *Escherichia coli* and *Klebsiella pneumoniae* among Iranain patients with inflammatory bowel disease (IBD)

**DOI:** 10.1186/s12941-023-00630-x

**Published:** 2023-09-14

**Authors:** Ayda Afshari Kharaghani, Naser Harzandi, Babak Khorsand, Mohsen Rajabnia, Azin Afshari Kharaghani, Hamidreza Houri

**Affiliations:** 1 Foodborne and Waterborne Diseases Research Center, Research Institute for Gastroenterology and Liver Diseases, Shahid Beheshti University of Medical Sciences, Tehran, Iran; 2 Department of Microbiology, Karaj Branch, Islamic Azad University, Karaj, Iran; 3 Gastroenterology and Liver Disease Research Center, Research Institute for Gastroenterology and Liver Diseases, Shahid Beheshti University of Medical Sciences, Tehran, Iran; 4 Non-Communicable Diseases Research Center, Alborz University of Medical Sciences, Karaj, Iran; 5 Foodborne and Waterborne Diseases Research Center, Research Institute for Gastroenterology and Liver Diseases, Shahid Beheshti University of Medical Sciences, Shahid Arabi Ave., Yemen St, Velenjak, Tehran Iran

**Keywords:** Inflammatory bowel disease, Ulcerative colitis, Crohn’s disease, Extended-spectrum β-lactamase, Enterobacterales, *Escherichia coli* and *Klebsiella pneumoniae*

## Abstract

**Background:**

Several pieces of evidence suggest that certain pathobionts belonging to Enterobacterales are associated with the development and progression of inflammatory bowel diseases (IBD). Extended-spectrum β-lactamases (ESBLs) ESBLs are frequently found in the Enterobacterales members, particularly in *Escherichia coli* and *Klebsiella* spp., and might trigger antibiotic-induced perturbations of the intestinal microbiota and led to more severe disease activity in IBD. Therefore, the severity of IBD could be influenced by ESBL-producing Enterobacterales, and hence, this study aimed to investigate the presence of ESBLs and carbapenemases among mucosa-associated *E. coli* and *Klebsiella pneumoniae* isolated from colonic biopsies of Iranian patients with IBD.

**Methods:**

In this cross-sectional study, *E. coli* and *K. pneumoniae* were isolated from inflamed ileum and/or colon tissue of patients with IBD, including Ulcerative colitis (UC) and Crohn’s disease (CD), during colonoscopy. Demographic data and clinical characteristics were recorded, and UC and CD disease activity and extent were evaluated according to the full Mayo score and Crohn’s disease activity index (CDAI), respectively. Phenotypic and molecular detection of ESBL- and carbapenemase-producing *E. coli* and *Klebsiella pneumoniae* were carried out. Disease activity and other clinical and microbial features were compared in patients with and without gut colonization with ESBL producers.

**Results:**

A total of 83 IBD patients, including 67 UC and 16 CD, were enrolled in the initial analysis. Intestinal colonization with ESBL-producing *E. coli* and/or *Klebsiella pneumoniae* was found in 37 (55.2%) of UC and 9 (56.2%) of DC patients – mostly harbored *E. coli* containing the *bla*_CTX−M_ and *bla*_TEM_ genes. UC patients with intestinal colonization with ESBL-producers had more severe disease compared with patients without colonization. Moreover, 10.2% of tested *E. coli* and 34.8% of *K. pneumoniea* were recognized as potential carbapenemase producers.

**Conclusion:**

Intestinal colonization with ESBL producers could arise disease activity in IBD patients. Further large-scale case-control studies should be performed to investigate the possible confounding factors that could contribute to this outcome.

## Background

Ulcerative colitis (UC) and Crohn’s disease (CD) are two types of inflammatory bowel diseases (IBD) that are chronic, progressive, and relapsing immune-mediated gut disorders of unknown etiology [1]. UC characteristically affects the rectum and generally extends proximally along the entire colon and can be classified as proctosigmoiditis, left-sided colitis, or pancolitis [2]. Bloody diarrhoea and tenesmus are the most common symptoms of UC, although it can be accompanied by extraintestinal complications due to intestinal inflammatory activity [3]. By contrast, CD is characterized by transmural inflammation and patchy lesions that are potentially scattered anywhere in the digestive tract leading to fibrosis, stricture, and fistula [4]. CD-related clinical manifestations are heterogeneous, but mainly include chronic abdominal pain, clinical signs of bowel obstruction and/or diarrhea with the passage of blood or mucus, and weight loss [5].

Etiological factors including genetic variances, immune response dysregulation, intestinal microbial alternations, and environmental changes have been described to play critical roles in the pathogenesis of IBD [6]. It has been long believed that the altered host-microbe homeostasis, defined as microbial “dysbiosis”, contributes to the pathogenesis and progression of IBD. Several pieces of evidence suggest that more severe CD and UC activity is associated with the Enterobacterales “blooming” following the disturbed microbiota in the gut [7]. Moreover, antibiotic-induced perturbations of the intestinal microbial community have long been described as a potential trigger for IBD [8].

Extended-spectrum β-lactamases (ESBLs) are bacterial enzymes that confer resistance to broad-spectrum β-lactam antibiotics including third-generation cephalosporins, and their presence is also frequently associated with resistance to other antimicrobial agents such as fluoroquinolones and aminoglycosides [9, 10]. ESBLs are frequently found in the Enterobacterales members, particularly in *Escherichia coli* and *Klebsiella* spp. which are the most common multidrug-resistant microorganisms (MDROs) found in the gut. More importantly, ESBL-related genes can be spread through the intestinal bacteria and enable the propagation of resistance to susceptible commensal strains in the gut. Vaisman et al. previously reported that one-third of hospitalized IBD patients who developed pouchitis were colonized with ESBL-producing Enterobacterales [11]. Moreover, Ananthakrishnan and McGinley found that more than one-quarter of hospitalizations for comprised CD and UC are associated with antibiotic-resistant infections which had four-fold greater mortality [12]. Therefore, the severity of the course of IBD may be influenced by ESBL-producing gut bacteria, and hence, this study aimed to investigate ESBL- and carbapenemases-producing *E. coli* and *Klebsiella pneumoniae* isolated from colonic biopsies of Iranian patients with IBD.

## Methods and materials

### Patients and sample collection

This study was carried out under the approval of the Ethical Review Committee of the Research Institute for Gastroenterology and Liver Diseases affiliated with Shahid Beheshti University of Medical Sciences, Tehran, Iran (Project No. IR.SBMU.RIGLD.REC.1401.010). Informed consent was obtained from all patients and/or their legal guardians before sample collection. In this cross-sectional study, patients with IBD, including CD or UC, who were referred to Taleghani hospital in Tehran, Iran were enrolled between July 2021 and August 2022. Diagnosis and classification of IBD depended on a combination of clinical, radiological, endoscopic, and pathological features [13]. All biopsy specimens were taken from inflamed tissue from the ileum and/or colon of IBD patients during colonoscopy. Demographic data and clinical characteristics were recorded for all patients through a questionnaire. The disease activity of UC and CD patients was evaluated using Mayo scoring and Crohn’s disease activity index (CDAI), respectively.

### Bacterial isolation and identification

Biopsy samples were taken with sterile forceps passed through the colonoscopy, placed in sterile tubes containing brain heart infusion (BHI) broth, and then immediately delivered to the microbiology laboratory of the Foodborne and Waterborne Diseases Research Center, Research Institute for Gastroenterology and Liver Diseases. The fresh biopsies were aseptically homogenized and cultured on MacConkey agar followed by overnight incubation at 37◦C in aerobic conditions. Subsequently, suspected lactose fermenting colonies with different colony morphology per sample were selected and subcultured for further bacterial verification. Identification of *E. coli* and *K. pneumoniae* isolates was performed based on conventional biochemical tests using oxidase, citrate utilization, urease production, methyl red, Voges-Proskauer, motility, and various sugar fermentation tests. Additionally, molecular confirmation was performed by PCR amplification of *phoA* gene of *E. coli* and *khe* gene in *K. pneumoniae* using specific primers as previously described [14].

### Antimicrobial susceptibility determination

Antimicrobial susceptibility of *E. coli* and *K. pneumoniae* isolates was carried out using the Kirby–Bauer disk diffusion method as recommended by the Clinical and Laboratory Standards Institute (CLSI) guidelines [15]. Commercially available antibiotic disks (Mast Co., United Kingdom) used in this study included ampicillin, (10 µg), piperacillin (100 µg), piperacillin-tazobactam (100/10 µg), cefoxitin (30 µg), ceftriaxone (30 µg), cefotaxime (30 µg), ceftazidime (30 µg), cefepime (30 µg), cefixime (30 µg), imipenem (10 µg), ertapenem (10 µg), meropenem (10 µg), aztreonam (30 µg), ciprofloxacin (5 µg), levofloxacin (5 µg), ofloxacin (5 µg), moxifloxacin (5 µg), gatifloxacin (5 µg), gentamicin (10 µg), amikacin (30 µg), kanamycin (30 µg), tobramycin (10 µg), tetracycline (30 µg), and trimethoprim/sulfamethoxazole (1.25/23.75 µg).

### Phenotypic screening for ESBLs and carbapenemase

*E. coli* and *K. pneumoniae* isolates that showed a reduced zone size to third-generation cephalosporins were selected for ESBL activity screening using the double disc synergy (DDT) following the procedure described by the CLSI. Accordingly, a ceftazidime disc (30 µg) was placed on the inoculated Mueller Hinton agar 15 mm away from the center of the amoxicillin-clavulanic acid disc (20 µg/10 µg). Extension of the zone of inhibition towards amoxicillin-clavulanic acid was interpreted as ESBL producer. Additionally, the Carba NP test, which has been described to be a highly sensitive method for the detection of *K. pneumoniae* carbapenemase (KPC) and metallo-beta-lactamase (MBL) [16], was performed to screen carbapenemase producers following the protocol described previously [17].

### Molecular detection of plasmid genes associated with ESBLs and carbapenemase

The genomic DNA was isolated from mucosa-associated *E. coli* and *K. pneumoniae* isolates from IBD patients using QIAamp DNA Mini Kit (QIAGEN, Hilden, Germany) according to the manufacturer’s instructions. Molecular detection of ESBLs was performed for the *bla*_TEM_, *bla*_SHV_, *bla*_CTX−M_, *bla*_PER_, *bla*_VEB_, and *bla*_GES_ genes using multiplex PCR assays as described previously with slight modifications [18]. Amplification was carried out as follows: initial denaturation at 94 °C for 10 min; 30 cycles of 94 °C for 40 s, 60 °C for 40 s, and 72 °C for 1 min; and a final elongation step at 72 °C for 7 min. To identify carbapenemase encoding genes, multiplex PCR assays were carried out for the amplification of *bla*_IMP_, *bla*_VIM_, *bla*_NDM_, *bla*_SPM_, *bla*_DIM_, and *bla*_KPC_ using specific primers as described previously [19]. The PCR products were separated into 1.5% agarose gels and visualized under UV light after the gels were stained with ethidium bromide.

### Statistical analysis

All data were assessed using R software (version 4.0.3, The R Foundation for Statistical Computing; Vienna, Austria). The correlation between variables was assessed using Spearman and Pearson’s correlation tests. Logistic regression analysis was applied to determine whether ESBL carriage is independently associated with the severity of IBD, or whether this is simply driven by the more expansive antibiotic treatment history in patients with more severe disease. All *P*-values were considered significant at a 5% level.

## Results

### Clinical characteristics and patients demographics

A total of 83 patients with confirmed IBD diagnosis, including 67 (80.7%) with UC and 16 (19.3%) with CD, were enrolled in the study. Based on colonoscopy and pathology findings, about 46.3% of UC patients had proctitis at diagnosis, followed by left-sided colitis (28.3%), backwash ileitis (16.4%), and pancolitis (9%). The majority of CD patients (62.5%) predominantly had ileal involvement, followed by the involvement of both ileum and colon (ileocolonic) (25%) and right-sided colitis (12.5%). Patients with UC had a median Mayo score of 9 (range 3–12) and in patients with CD, the median baseline CDAI was 129 (range 56–348). In this study, a Mayo score of 3 to 5 points indicates mildly active disease, a score of 6 to 9 points indicates moderately active disease, and a score of 10 to 12 points indicates severely active disease in UC patients. Accordingly, a CDAI score of 150–219 has been labeled as a mildly active disease and 220–400 as a moderately active disease in CD patients. No CD patients had severely active disease based on CDAI scoring. About one-third of IBD patients had a history of prior antibiotic therapy with beta-lactams (31.3%) or quinolones (38.5%). Details of the demographic and clinical characteristics of the patients are summarized in Table 1.

**Table 1:** Baseline demographic and characteristics of patients with IBD enrolled in this study

Variable	Crohn’s disease *n* = 16	Ulcerative colitis *n* = 67
**Age, median [years](± IQR)**	34.5 (± 20.25)	35 (± 15.25)
**BMI, mean [kg/m²] (± SD)**	33.54 (± 6.2)	24.17 (± 5.9)
**Male, n (%)**	10 (62.5%)	39 (58.2%)
**Smoking, n (%)**	6 (37.5%)	17 (25.3%)
**Alcohol use, n (%)**	1 (6.2%)	7 (10.4%)
**Drug abuse, n (%)**	1 (6.2%)	4 (6%)
**Familial history of IBD, n (%)**	2 (12.5%)	5 (7.5%)
**Complications, n (%)**		
Diarrhea	12 (75%)	51(76.1%)
Bloody diarrhea	7 (43.7%)	38 (56.7%)
Constipation	1 (6.2%)	3 (4.5%)
weakness	2 (12.5%)	12 (17.9%)
Weight loss	3 (18.7%)	19 (28.3%)
Hematochezia	0 (0)	10 (14.9%)
Fever	0 (0)	2 (3%)
Nausea and Vomiting	0 (0)	12 (17.9%)
**Laboratory results, median (± IQR)**		
WBC, ×10^9^/L	9.1 (± 0.5)	5.9 * 10^3^ (± 5)
Hemoglobin, g/dL	12.21 (± 4.14)	10.7 (± 5)
Platelets, ×10^9^/L	244.5 (± 18.75)	261 (± 81)
MCV, femtoliter	83.3 (± 8.45)	92.8 (± 14.4)
ESR, mm/hr	24 (± 21.75)	51 (± 16.75)
CRP, mg/dL	75 (± 44.9)	62.5 (± 36.75)
Total bilirubin, mg/dL	0.65 (± 0.37)	0.7 (± 1.15)
Direct bilirubin, mg/dL	0.2 (± 0.7)	0.2 (± 0.35)
AST, U/L	23 (± 9.75)	26 (± 56)
ALT, U/L	18 (± 6.5)	23 (± 43)
ALP, U/L	165.5 (± 37)	173 (± 100)
BUN, mg/dL	15 (± 9)	15 (± 7)
**Medications, n (%)**		
β-lactams	5 (31.2%)	21 (31.3%)
Fluoroquinolones	7 (43.7%)	25 (37.3%)
Metronidazole	7 (43.7%)	23 (34.3%)
NSAIDs	7 (43.7%)	37 (55.2%)
SID	1 (6.2%)	14 (20.8%)
PPI	4 (25%)	12 (17.9%)
Anti-TNF biologics	6 (37.5%)	29 (43.3%)
**Disease activity index, median (range)**		
Mayo Score	-	9 (3–12)
CDAI	129 (56–348)	-

CDAI, Crohn’s disease activity index; PPI, proton-pump inhibitors; NSAIDs, non-steroidal anti-inflammatory drugs; SID, corticosteroid immunosuppressive drugs

### Bacterial isolates and antibiotic resistance profiles

**Fig. 1:**
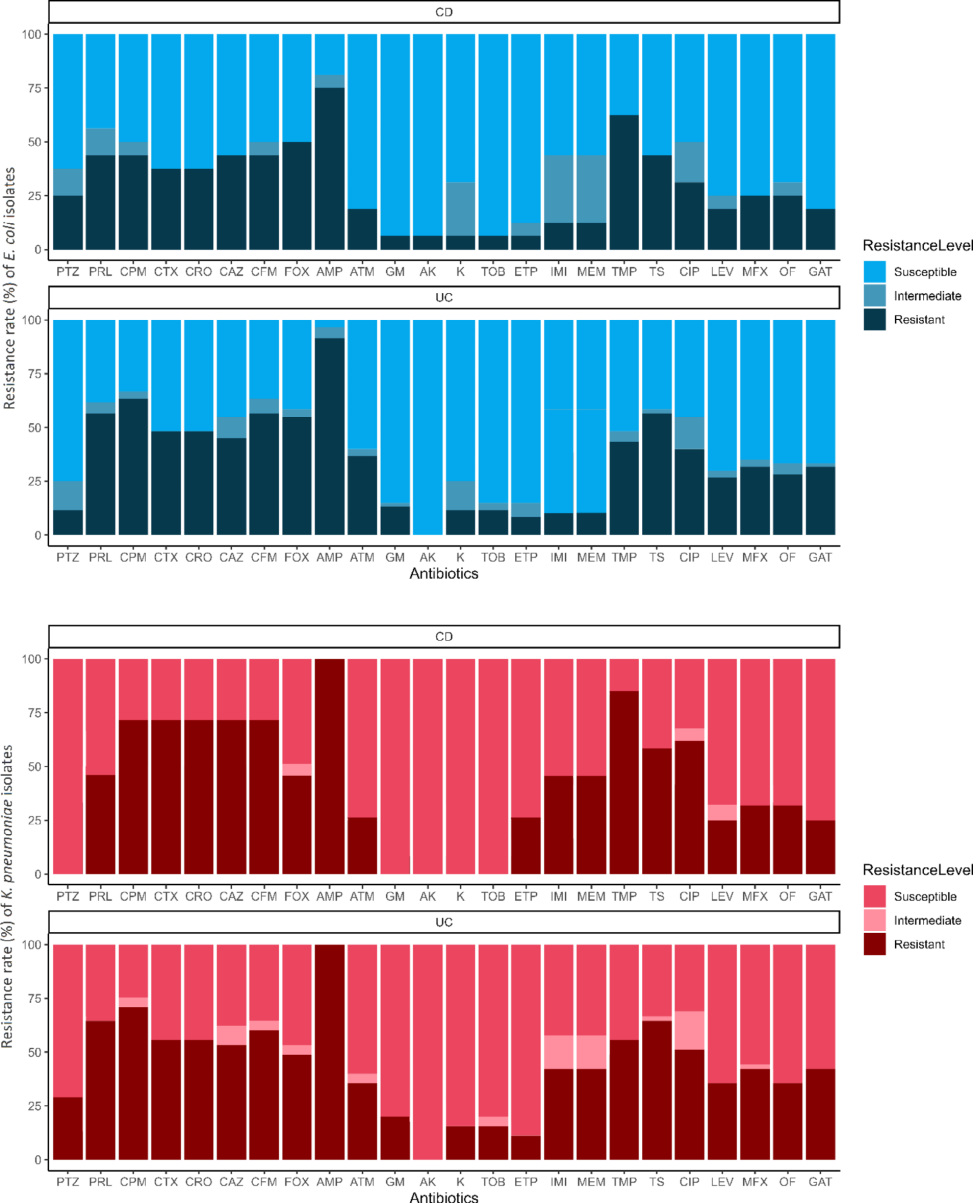
Frequency bar chart illustrating phenotypic antibiotic susceptibility profiles of mucosa-associated *E. coli* (blue chart) and *K. pneumoniea* (red chart) isolates from colonic biopsies of patients with IBD. Abbreviations: AK, amikacin; AMP, ampicillin; ATM, aztreonam; CAZ, ceftazidime; CFM, cefixime; CIP, ciprofloxacin; CPM, cefepime; CRO, ceftriaxone; CTX, cefotaxime; ETP, ertapenem; FOX, cefoxitin; GAT, gatifloxacin; GM, gentamicin; IMI, imipenem; K, kanamycin; LEV, levofloxacin; MEM, meropenem; MFX, moxifloxacin; OF, ofloxacin; PRL, piperacillin; PTZ, piperacillin-tazobactam; TMP, trimethoprim; TOB, tobramycin; TS, trimethoprim-sulfamethoxazole

In this study, 68 mucosa-associated *E. coli* (54 from UC and 14 from CD) and 23 mucosa-associated *K. pneumoniea* (16 from UC and 7 from CD) isolates were recovered from biopsies of IBD patients, in which some patients harbored different strains. As shown in Fig. 1, antimicrobial susceptibility testing revealed that the majority of *E. coli* and *K. pneumoniea* isolates were non-susceptible to ampicillin, cefoxitin, cefepime, and trimethoprim, and 56% (51/91) of the isolates considered MDR, including 55.7% (39/70) of UC- and 57.2% (12/21) of CD-associated isolates.

### Characteristics of ESBL- and carbapenemase-producing isolates

**Fig. 2:**
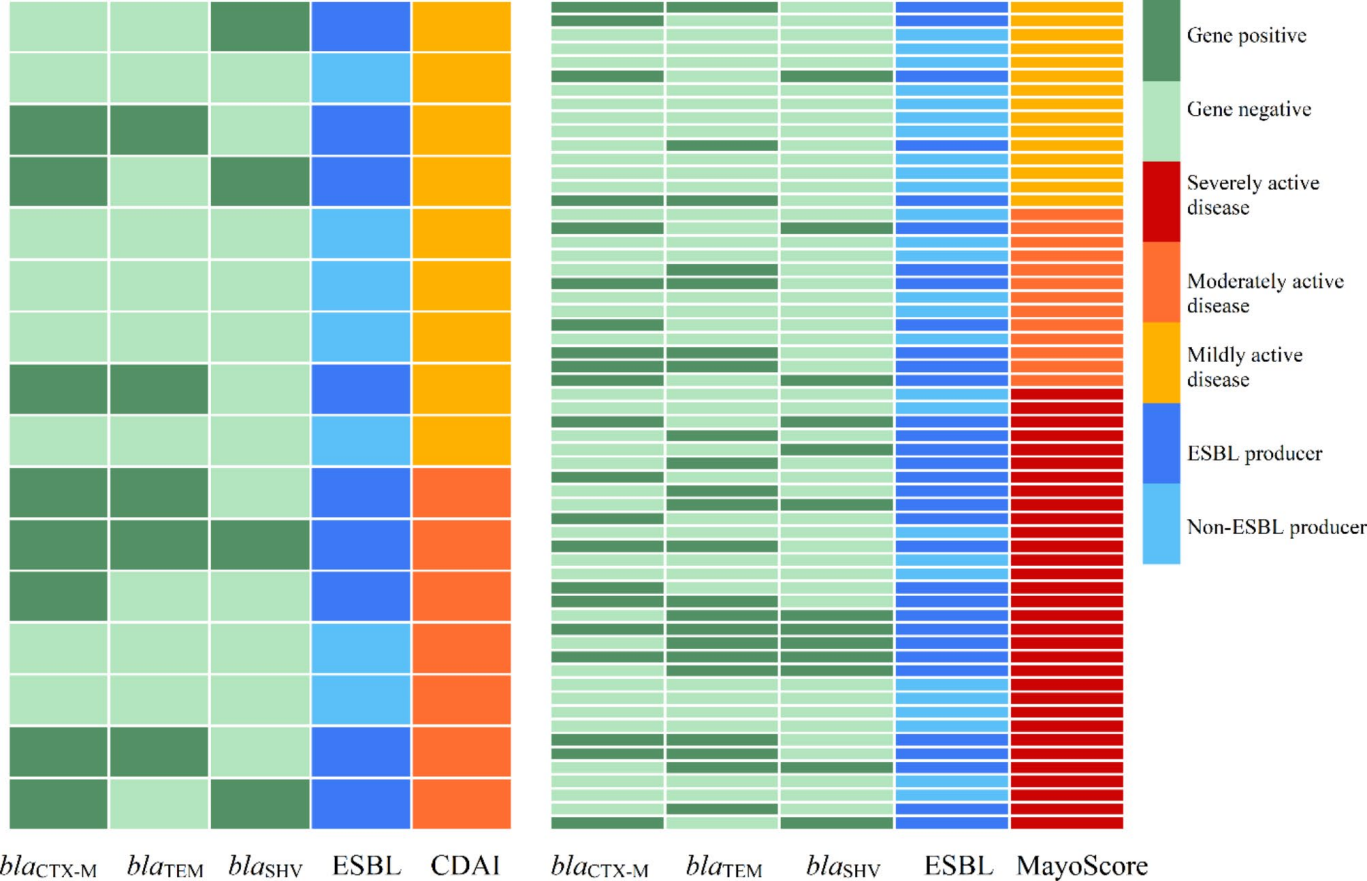
Distribution of ESBL-producing *E. coli* and/or *K. pneumoniea* isolates and three major related determinants among patients with CD (left) and UC (right) according to their disease severity (e.g., CDAI or Mayo scoring) Abbreviations: CDAI, Crohn’s disease activity index

**Fig. 3:**
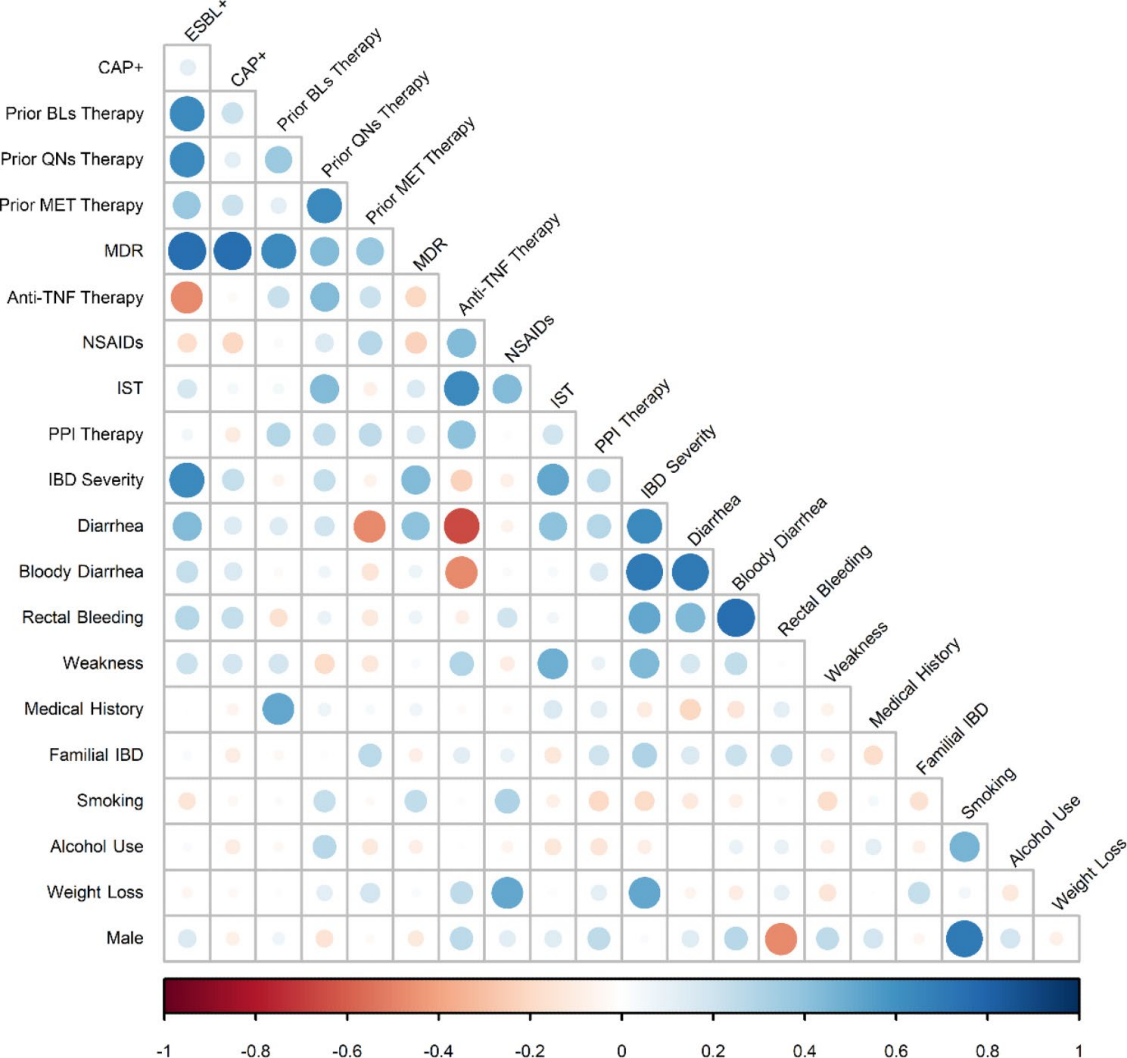
Heatmap illustrating the pairwise correlation between bacterial isolates as well as clinical and demographic features of patients with IBD calculated with Pearson’s correlation. The bigger the circle and the deeper the color represents a higher correlation Abbreviations: BLs, beta-lactams; CAP+, carbapenemase-producers; ESBL+, ESBL-producers; IST, immunosuppressant therapy with steroids; MDR, multi-drug resistance; MET, metronidazole; NSAIDs, nonsteroidal anti-inflammatory drugs, PPI, proton-pump inhibitor drugs; QNs, quinolones

Phenotypic screening for ESBLs indicated that 54.4% (37/68) of investigated mucosa-associated *E. coli* and 69.6% (16/23) of *K. pneumoniea* isolates showed ESBL activity. Indeed, gut colonization with ESBL-producing *E. coli* and/or *K. pneumoniae* strains was found in 55.2% (37/67) of UC and 56.2% (9/16) of DC patients. Moreover, 7.3% (5/68) of tested *E. coli* and 34.8% (8/23) of *K. pneumoniea* were recognized as potential carbapenemase producers. Molecular analysis confirmed the presence of *bla*_CTX−M_, *bla*_SHV_, *bla*_TEM_, and *bla*_VEB_ ESBL genes, in which *bla*_CTX−M_ and *bla*_TEM_ were the most prevalent ESBL genes among mucosa-associated *E. coli* (39.9% and 31.4%) and *K. pneumoniea* (42.8% and 21.4%), respectively. In addition, unfortunately, carbapenemase-encoding genes *bla*_NDM_, *bla*_IMP_, and *bla*_KPC_ were detected in *E. coli* and *K. pneumoniea* isolates. Table 2 summarizes the frequency of ESBL- and carbapenemase-associated mobile genetic elements detected in mucosa-associated *E. coli* and *K. pneumoniea* isolates from UC and CD patients. Figure 2 shows a schematic illustration that represents the distribution of ESBL-producing *E. coli* and/or *K. pneumoniea* isolates, as well as three major related ESBL genes, among patients with IBD according to their disease severity, e.g., CDAI or Mayo scoring. According to Pearson’s correlation coefficient analysis, a significant positive correlation was found between the intestinal carriage of ESBL-producing *E. coli* and/or *K. pneumoniea* and the disease severity (based on Mayo scoring) in the patients with UC (*P*-value = 0.03), indicating more severe patients were more likely to be colonized with ESBL producers (Fig. 3). In addition, there was a significant positive correlation between the presence of ESBL-harboring strains and prior antibiotic therapy with β-lactams and/or quinolones (*P*-value = 0.01). Additionally, mucosa-associated ESBL- and carbapenemase-producing isolates were more likely to be MDR (*P*-value = 0.01). Figure 3 is a heatmap representing the correlation between the presence of ESBL-producing *E. coli* and *K. pneumoniea* isolates and the clinical and demographic features of patients with IBD enrolled in this study.

**Table 2:** Frequency of ESBL- and carbapenemase-associated mobile genetic elements among mucosa-associated *E. coli* and *K. pneumoniea* isolates from colonic biopsies of patients with IBD.

ESBL genes	IBD-related isolates				
	*E. coli* (MDR) N = 68		* K. pneumoniae* (MDR) N = 23		
	*n*	%		*n*	%
*bla*_CTX−M_ (*n* = 32)	27 (17)	39.7% (25%)		5 (4)	21.7% (17.3%)
*bla*_SHV_ (*n* = 17)	11 (6)	16.1% (8.8%)		6 (5)	26% (21.7%)
*bla*_TEM_ (*n* = 29)	20 (11)	29.4% (16.1%)		9 (8)	39.1% (34.7%)
*bla*_VEB_ (*n* = 1)	1 (1)	1.8% (1.8%)		-	-
*bla*_GES_ (*n* = 0)	-	-		-	-
*bla*_PER_ (*n* = 0)	-	-		-	-
**CAP genes**					
*bla*_NDM_ (*n* = 2)	1 (1)	1.8% (1.8%)		2 (2)	12.5% (12.5%)
*bla*_KPC_ (*n* = 2)	-	-		3 (3)	13% (13%)
*bla*_DIM_ (*n* = 0)	-	-		-	-
*bla*_IMP_ (*n* = 2)	1 (1)	1.8% (1.8%)		1 (1)	6.2% (6.2%)
*bla*_VIM_ (*n* = 0)	-	-		-	-
*bla*_SPM_ (*n* = 0)	-	-		-	-

Abbreviations: CAP, carbapenemase; CD, Crohn’s disease; ESBL, extended-spectrum beta-lactamases; MDR, multidrug resistant; UC, ulcerative colitis

**Fig. 4:**
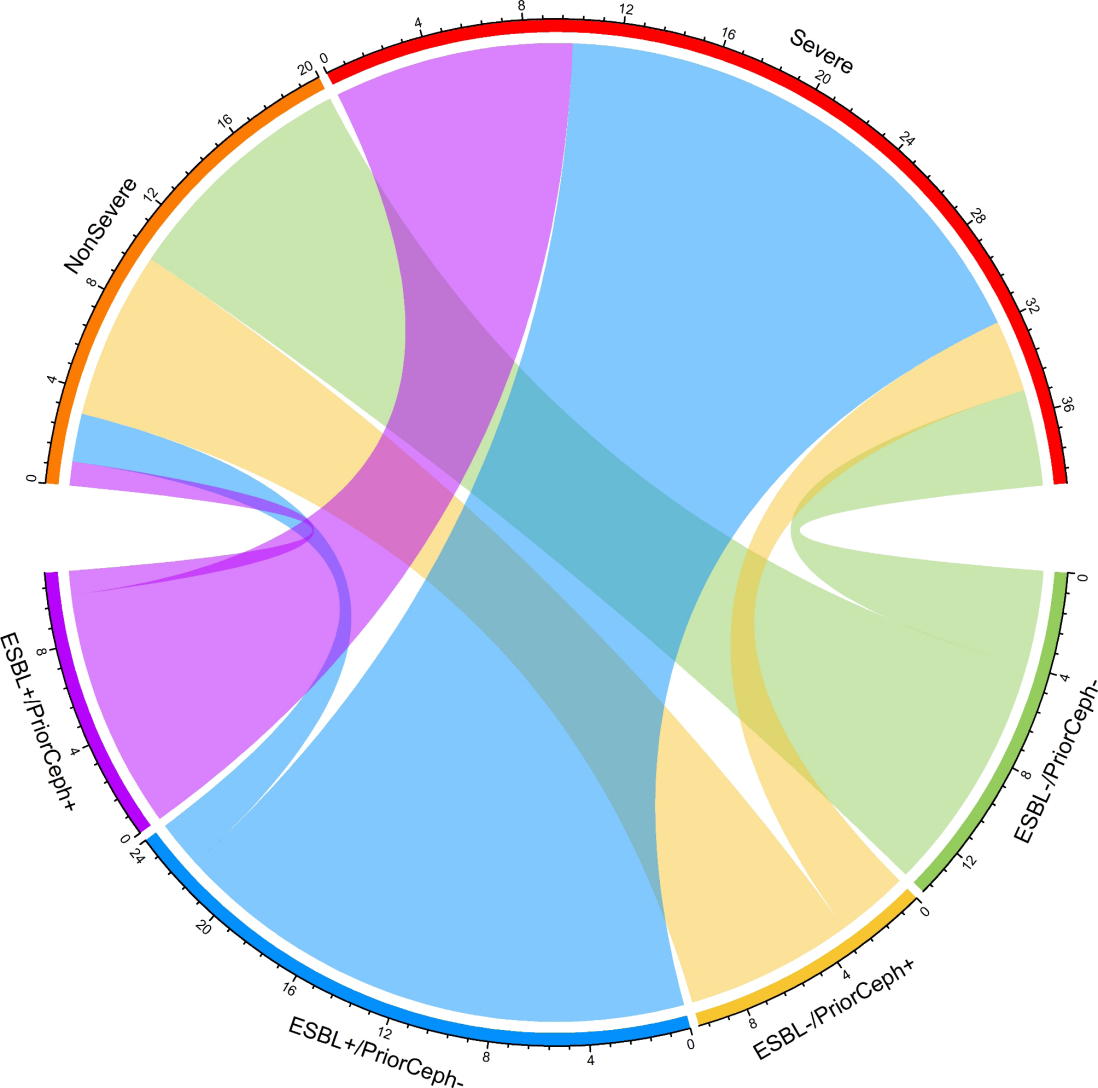
Chord-diagram visualizing the correlation between disease severity and ESBL carriage, taking into account the history of antibiotic treatment in patients with UC. Our findings revealed that the severity of the disease was linked to the presence of ESBL producers, regardless of whether UC patients had received long-term antibiotic treatment prior to that Abbreviations: ESBL+, carriage of ESBL-producers; ESBL-, carriage of ESBL non-producers; PriorCeph+, patients had a history of antibiotic treatment with cephalosporins; PriorCeph-, patients had no history of long-term exposure to cephalosporins previously

To determine if carrying ESBL-producer bacteria is linked to the disease severity independently or if it is influenced by a more extensive history of antibiotic treatment in patients with severe disease, we categorized our main outcome variable into two groups: severe disease and non-severe. We then conducted binary logistic regression to examine which input variables are associated with the patient’s outcome. Accordingly, our analysis revealed that ESBL carriage was found to be associated with disease severity among patients with UC (odds ratio (OR) 4.25 [95% CI 2.76 to 5.73], *P*-value = 0.001), however, there was no significant association between prior antibiotic therapy and disease severity (odds ratio (OR) 1.01 [95% CI 0.26 to 1.75], *P*-value = 0.02). To enhance comprehension, we have additionally depicted the relationship between the severity of the disease and the carriage of ESBL producers, taking into account the history of long-term antibiotic treatment with beta-lactams, as illustrated in Fig. 4.

## Discussion

Changes in the gut microbiome with an expansion of certain potentially harmful proteobacteria, particularly pathobiont strains belonging to the Enterobacterales, have been described to be associated with increased disease activity and a higher risk of treatment failure in patients with IBD [20–22]. Furthermore, asymptomatically intestinal colonization with ESBL-producing Enterobacterales results not only in the spread of dangerous MDROs and superbugs but also in the favor of the reduction in microbial richness and diversity and the overgrowth of Proteobacteria and Enterobacterales pathobionts, particularly of drug-resistant *E. coli* and *K. pneumoniea* strains [23]. Moreover, it has been previously reported that ESBL-producing Enterobacterales carriers may suffer from sub-acute intestinal inflammation leading to gut microbiome dysbiosis [23]. With this in mind and based on the fact that *E. coli* and *K. pneumoniea* are the most common ESBL-producing bacteria colonizing the intestinal tract, we hypothesized that patients with IBD colonized intestinally with ESBL-producing isolates may have a more severe disease course. To the best of our knowledge, the prevalence of intestinal colonized ESBL-producing Enterobacterales has never been reported in an Iranian IBD patient population before, while these superbugs have a relatively high prevalence among Iranian population in both healthcare settings and communities [24, 25]. This is the first study to investigate the carriage status of mucosa-associated ESBL-producing *E. coli* and *K. pneumoniea* among Iranian patients with IBD.

Notoriously, we found that more than half of IBD patients were intestinally colonized with ESBL-producing *E. coli* and/or *K. pneumoniea*, which was five times more frequently than described in the European [26], Canadian [11, 27], and Chinese [27] IBD patient population. Moreover, ESBL producers in our study were significantly associated with the MDR phenotype, which is in accordance with previous reports [28, 29]. As IBD patients have a significantly elevated risk of bacterial infection that is primarily a consequence of the wide use of immunomodulators and increased intestinal permeability [30], the high colonization rate with ESBL-producers and MDROs could potentially enhance overall mortality, morbidity, and economic burden attributable to IBD-related complications. Notably, to our knowledge, this study is the first to report the intestinal carriage of *bla*_NDM_, *bla*_KPC_, and *bla*_IMP_-harboring *E. coli* and *K. pneumoniae* among patients with IBD. A matter of concern is that the enrichment of such intestinal pathobionts has been described to be associated with increasing the permeability of the intestinal epithelial barriers and facilitating the translocation of the bacteria to the bloodstream [31, 32], and it may be more considerable in individuals with intestinal barrier dysfunction like IBD patients. The empirical antimicrobial therapy of infections with carbapenemase producers is not precisely addressed by infection disease societies such as the Infectious Diseases Society of America (IDSA) and the European Society of Clinical Microbiology and Infectious Diseases (ESCMID), and available treatments are limited with a significant ecological impact and possible toxicity [33, 34]. However, although intestinal carriage of carbapenemase producers may contribute to the development of severe life-threatening infections and increase the risk of comorbidities [35], we could not describe significant risk factors for these infections in carriers with IBD and further research efforts are needed to obtain more conclusive considerations in this regard.

Remarkably, we found that UC patients colonized with ESBL producers tended to have greater disease severity and symptoms than non-carriers. These observations are in accordance with those previously reported by Skujaa et al. [26], in which UC patients with ESBL-producing Enterobacterales gut colonization exhibited worse disease activity. Distinctively, we analyzed our data to determine whether ESBL producers are being selected by the regular and long-term exposure to antimicrobial agents (especially β-lactams) in more severe patients during hospitalization, infectious complications, and surgeries, or whether the colonization with ESBL-producing *E. coli* and/or *K. pneumoniea* increases the risk of the overgrowth of either resident or introduced pathobionts. According to our multivariate analysis, ESBL carriage was independently associated with the disease severity in UC patients, and this correlation was not dependent on prior long-term antibiotic treatment in patients with more severe disease. As described previously, antibiotic-resistant Enterobacterales pathobionts ‘blooms’ are associated with more severe intestinal inflammation and colitis [22, 36, 37].

We found a positive correlation between the presence of ESBL-harboring *E. coli* and/or *K. pneumoniea* strains and prior antibiotic therapy with β-lactams and/or quinolones. It is a widespread opinion that prior receipt of third-generation cephalosporins is a risk factor for the acquisition of ESBL-producing Enterobacterales [38]. Not surprisingly, most of the IBD patients harboring ESBL producers in this study had a history of long-term antibiotic therapy with third-generation cephalosporins, including cefixime or ceftriaxone. Furthermore, emerging resistance to ciprofloxacin, the antibiotic of choice in complicated IBD cases, has been reported in ESBL-producing Enterobacterales colonizing the gut [39, 40]. Our results provide evidence of ciprofloxacin resistance in more than 70% of ESBL-producing *E. coli* and/or *K. pneumoniea* isolates from IBD patients. Accordingly, a high gut colonization rate with ESBL-carrying Enterobacterales, mainly *E. coli* strains expressing the CTX-M gene, in UC patients was formerly found to be associated with high resistance to ciprofloxacin by Skuja et al. [41]. These observations imply that the relationship between ESBL production and ciprofloxacin resistance in such cases may be due to the interplay between long-term broad-spectrum antibiotic therapy and conditions favoring the appearance of MDROs. An assist conceivable clarification for the co-occurrence of ESBL production with ciprofloxacin resistance is transporting the resistance determinates on the same plasmids [42]. Moreover, It has been documented that ESBL-producing bacteria tend to multiply resistant against other broad-spectrum antimicrobial agents [43].

The role of antimicrobial therapy in IBD is controversial and there is currently insufficient data to recommend antibiotic therapy or not. Based on the role of gut aerobic and anaerobic pathobionts in the progression of IBD, antimicrobial combination therapy appears to be a rational strategy in the management of the primary disease process or its complications. Accordingly, using ciprofloxacin combined with metronidazole is widely accepted for the treatment of colitis and perianal fistula in severe cases of UC and CD. However, on the other hand, there is a considerable risk for intestinal overcolonization with multidrug-resistant pathobionts due to the antibiotic selective pressure in IBD patients during empirical antimicrobial therapy. Dubinsky et al. have recently reported that long-term antibiotic treatment used to alleviate pouchitis in patients with UC could enrich the strains that acquired multidrug resistance loci, including ESBLs [44]. Hence, non-empirical antimicrobial and combined therapies are suggested for severe cases of IBD colonized by resistant strains. In addition, the common use of broad-spectrum antimicrobial agents is not recommended in mild or moderate cases of IBD [45].

## Conclusions

In summary, a high prevalence of mucosa-associated ESBL-producing *E. coli* and/or *K. pneumoniea* in IBD patients obtained in this study should be taken into consideration in the implementation of antimicrobial selection by gastroenterologists in these patients as well as infection control experts. Moreover, given our data, colonization with ESBL producers could arise disease activity in these patients. Therefore, broad-spectrum antimicrobial therapy may be a double-edged sword, and hence, further large-scale randomized clinical trials should be carried out to explore the effect of antibacterial therapy, either individually or combined with other agents or therapies, in the management of IBD. In addition, determining the antibiotic susceptibility of fecal *E. coli* and/or *K. pneumoniea* may be useful for selecting appropriate treatments for patients overcolonized by these pathobionts or recognizing therapies that may pose a potential risk.

## Data Availability

All data generated or analysed during this study are included in this published article.

## Abbreviations

BHI: Brain heart infusion
CD: Crohn’s disease
CDAI: Crohn’s disease activity index
ESBL: Extended-spectrum β-lactamase
IBD: Inflammatory bowel diseases
MDROs: Multidrug-resistant microorganisms
UC: Ulcerative colitis
